# A Single Centre Experience of Day Case Laparoscopic Cholecystectomy Outcomes by Body Mass Index Group

**DOI:** 10.1155/2017/1017584

**Published:** 2017-09-28

**Authors:** Kirk Bowling, Samantha Leong, Sarah El-Badawy, Erfan Massri, Jaideep Rait, Jay Atkinson, Gandrapu Srinivas, Stuart Andrews

**Affiliations:** ^1^Peninsula Deanery, Torbay Hospital, South Devon Healthcare Trust, Lawes Bridge, Torquay TQ2 7AA, UK; ^2^Peninsula Deanery, Derriford Hospital, Plymouth Healthcare Trust, Derriford Road, Plymouth PL6 8DH, UK

## Abstract

**Aim:**

The purpose of this study was to evaluate whether patients with a high BMI can undergo safe day case LC for cholecystitis compared to groups of patients with a lower BMI.

**Setting:**

NHS District General Hospital, UK.

**Methods:**

A retrospective review of 2391 patients who underwent an attempted day case LC between 1 January 2009 and 15 August 2015 was performed. Patients were divided into five groups depending on their BMI. Inclusion criteria were patients undergoing elective day case laparoscopic cholecystectomy with cholecystitis on histology. The endpoints were complication requiring readmission and postoperative length of stay (LOS).

**Results:**

There were 2391 LCs performed in the time period of which 1646 were eligible for inclusion. These LCs were classified as 273 (16.9%), 608 (37.8%), 428 (26.6%), 208 (12.9%), and 91 (5.66%) patients in the groups with BMI values of 18.5–24.9, 25–29.9, 30–34.9, 35–39.9, and >40, respectively. Average BMI was 30.0 (±5.53, 19–51) with an average postoperative LOS of 0.86, and there was no difference between the BMI groups. Overall complication rate was 4.3%; there was no significance between BMI groups.

**Conclusions:**

Increased BMI was not associated with worse outcomes after day case LC.

## 1. Background

Laparoscopic cholecystectomy (LC) has become the standard of care for the treatment of symptomatic gallbladder disease [1]. Compared to the traditional open cholecystectomy (OC), LC is associated with lower morbidity and mortality, shorter length of hospital stay, and quicker return to normal activities [2].

Obesity in the United Kingdom is a growing problem and is one of the leading causes of preventable death in the UK. Adult obesity rates have almost quadrupled in the last 25 years, with 23.1% of British people obese as of 2012 and one-third of all UK males predicted to be obese by 2030 [3, 4].

Many studies have looked for factors that are associated with higher risks of conversion and complications. Several factors that have been identified are increased age, time of day, male gender, increased acuity of illness, and many others [5–8].

There is evidence that we aim to further add to that day case laparoscopic cholecystectomy can be performed safely in patients with a high BMI (Body Mass Index) without a higher readmission or complication rate; most of the current evidence is from the United States of America [9].

The British Association of Day Surgery already recommends that providing adequate training, equipment, and staff is present; patients with an increased BMI should be operated on in the day case setting. This is due to factors such as early mobilization and short anesthetics being of great benefit to these groups of patients in their recovery and that obesity per se is not a contraindication to day case surgery [10].

With increased prevalence of obesity and increasing experience with managing such patients, patients with a higher BMI are being operated on more routinely in the District General Hospital setting for day case laparoscopic cholecystectomy. However to the authors best knowledge that many units have different policies with regard to obesity in the day case unit.

Surgery in the obese has traditionally been labeled as high risk. We hypothesized that obesity may not be an independent significant risk factor leading to increased conversion, complication rates, and readmission.

With an increasing proportion of patients having a Body Mass Index (BMI) of more than 30 our study draws light on the impact of an increased BMI on the day case LC within the District General Hospital setting.

## 2. Aim

The primary outcome of this study is readmission rate following day case LC. Secondary outcomes include LOS (length of stay), conversion, complication rate, and mortality. These outcomes will be measured with the aim of concluding whether day case LC can be performed safely in patients with higher BMI's in a District General Hospital setting.

## 3. Methods

A retrospective review of a prospectively maintained database identified 2391 patients who underwent an attempted LC between 1 January 2009 and 15 August 2015. This database included standard demographical data such as height, weight, BMI, and patient identifiers. Each patient case was cross-referenced with the hospital episode statistics database and the theatre and pathology databases. This allowed compilation of data for each patient. Patients were excluded if classified as an emergency or if the indication was not gallstone disease. Inclusion and exclusion criteria can be seen in Table 1.

Cholecystitis was the main inclusion criteria as to decrease variability in the difficulty of the operation and decrease heterogeneity within the data.

Patients were divided into five groups depending on their BMI: 18.5–24.9, 25–29.9, 30–34.9, 35–39.9, and >40. The primary endpoints were conversion rates, complication rates, and postoperative length of stay. Complications were defined as any event requiring a procedure or hospital admission. Surgical site infections not requiring hospital admission were excluded. A hospital admission was any readmission to hospital 30 days after the procedure but did not include prolonged hospital stay from day case.

A small number of patients were identified as being readmissions but found to have illness separate to the original surgery. For example, a patient was readmitted for removal of a suspected melanoma electively.

Pearson Chi-Square and ANOVA tests were performed to check for statistical significance.

SPSS software version 19.0 (SPSS, Chicago, IL, USA) was used for statistical computation, and *P* < 0.05 was considered significant.

## 4. Results

**Figure 1:**
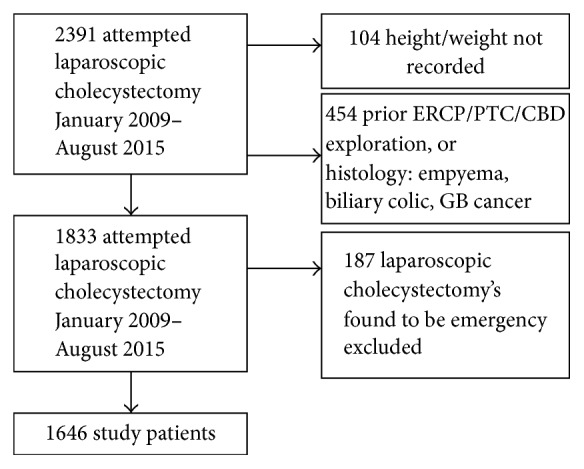
Study patients inclusion breakdown; CBD: Common Bile Duct; PTC: Percutaneous Transcutaneous Cholangiogram; ERCP: Endoscopic Retrograde Cholangiopancreatography.

There were 2391 LCs performed between 1 January 2009 and 15 August 2015; 2204 were elective nonemergency cases; 1646 cases were appropriate for study. See the following tables for inclusion criteria and excluded cases (Tables 1 and 2 and Figure 1).

These were distributed as per Table 3 into WHO (World Health Organization) recognized BMI groups.

Seven (0.44%) patients required conversion to open surgery. There was no significance for the rate of conversion amongst the BMI groups (*P* = 0.835) and postoperative LOS (*P* = 0.86). Overall complication rate was 4.3% including wound infections through to bile leaks (0.18%) again with no statistical significance between BMI groups (Table 4).

In Table 5 the readmission events are broken down by cause; the delineation between nonspecific chest pain and pain with no cause found classifications is an arbitrary one by the authors. The latter classification was made where a patient was readmitted for pain which was warranted serious enough for investigation in the form of imaging USS, CT, or CTPA (Table 5) with no positive finding and where the patient did not warrant intervention, the former being chest pain investigated and being refuted as cardiac or patients observed and then subsequently discharged with no cause found.

## 5. Discussion

The data showed an expected demographical distribution of patients; the majority of patients are female with a mean age of 53.4. However it is also shown that the average BMI of patients appears to be increasing with over 45% of all included LCs being performed on patients with class I obesity or above.

Within the category of small bowel obstruction all three patients required return to theatre for port site hernia repair with none requiring resection. All seven patients with retained stones were successfully managed with ERCP.

Despite nearly half of patients being obese there is no statistical significance between the groups in terms of conversion rate, complication rate, or LOS. However a proportion of patients were excluded from analysis due to no BMI value availability (*n* = 104, 5.67%). This is concerning with regard to the robustness of the data leading to selection bias. This could manifest in the form of patients whose weight was not recordable on the preassessment scales leading to patients with very high BMIs being excluded. In this excluded group no bile leaks were identified, or readmissions or deaths; it is therefore unlikely that the primary outcomes would be affected, but other outcomes could be influenced such as conversion rate.

Of note the data does not differentiate between the degree of cholecystitis, mainly due to the fact that there is no clear grading system of cholecystitis that could be applied to the data retrospectively other than chronic versus acute on histology. It was for this reason that biliary colic was excluded as although cholecystitis can lead to a variable difficulty in operation it was felt amongst the authors that this was acceptable and should not introduce bias to the data. Biliary colic inclusion, however, would lead to too much heterogeneity within the dataset.

Also comorbidities were not included in this study such as diabetes and steroid use. However these factors if managed appropriately should not affect day case management and indeed the guidelines from the British Association of Day Surgery state patients with such comorbidities are best managed in the day case setting.

Despite these limitations the mean LOS and secondary outcomes appear to not be affected by BMI category. Each individual readmission event derives no statistical significance by BMI grouping. Overall significant complication rates are shown to be low in our study and laparoscopic cholecystectomy is a safe procedure with BMI not being an independent risk factor for major complications.

## 6. Conclusion

The data corroborates demographical data from the Office of National Statistics that the patients we operate on are presenting with increased BMIs with only 17% of the patients having a normal BMI. This dataset offers a large sample size; however as mentioned in Discussion 5.67% of patients were excluded on the basis of no BMI data. Of this excluded group there were no bile leaks or deaths and these outcomes would unlikely be affected. However of the data available it shows clearly that increased BMI was not associated with statistically worse outcomes after day case LC. Compared with normal weight patients, obese and even morbidly obese patients have no increased risk of conversion to open surgery, or complications. Readmission rate and LOS are also not significantly influenced by BMI. This study therefore supports previous research and the British Association of Surgery guidelines that patients within an increased BMI class if managed appropriately have no worse outcomes than the normal BMI class if operated on in a District General setting with adequate training, staff, and equipment to handle such cases. It does not offer any evidence to the operative outcomes of obese patients in the emergency setting; this should be an area of further study. We therefore conclude that such patients can be managed without specialist bariatric input in the District General Hospital setting safely compared to other BMI groups in the elective day case setting effectively with appropriate staff, training, and equipment. However it is the authors opinion that an open discussion should take place with all patients who are eligible for specialist bariatric input with regard to the options available. As within our practice a number of patients select referral to a weight loss management service for a potential combined weight loss procedure and laparoscopic cholecystectomy; however this needs to be managed against patient symptoms and risk.

## Figures and Tables

**Table 1:** Inclusion and exclusion criteria.

Inclusion criteria	Exclusion criteria
(1) Day case laparoscopic cholecystectomy	(1) Emergency cholecystectomy
(2) Any BMI, any sex	(2) Planned open cholecystectomy
(3) Cholecystitis	(3) CBD exploration
	(4) Previous ERCP/PTC
	(5) Empyema, hepatobiliary cancer
	(6) No recorded BMI

BMI: Body Mass Index; CBD: Common Bile Duct; PTC: Percutaneous Transcutaneous Cholangiogram; ERCP: Endoscopic Retrograde Cholangiopancreatography.

**Table 2:** Patient demographics.

Total patients	1646
Male/female	354/1292
Age (year) mean ± SD, range	53.4 ± 16.25, 16–87
BMI (kg/m2) mean ± SD, range	30.0 ± 5.53, 19–51

SD: Standard Deviation; BMI: Body Mass Index.

**Table 3:** Distribution of patients by BMI groups.

Normal weight BMI 18.5–24.9	280 (17.0%)
Overweight BMI 25.0–29.9	620 (37.7%)
Class I obesity BMI 30.0–34.9	438 (26.6%)
Class II obesity BMI 35.0–39.9	213 (12.9%)
Class III obesity BMI > 40.0	95 (5.8%)
Total	1646

BMI: Body Mass Index.

**Table 4:** Table showing patient outcomes categorized by BMI group.

	Total	<24.9	25–29.9	30–34.9	35–39.9	>40	Sig
	(*n* = 1646)	(*n* = 280)	(*n* = 620)	(*n* = 438)	(*n* = 213)	(*n* = 95)	
Conversion	7 (4.3%)	2	3	1	1	0	0.835^*∗*^
Complication	65 (3.95%)	9	31	15	7	3	0.183^*∗*^
Mean LOS ± SD (days)	0.86	0.83 ± 2.20	0.88 ± 2.22	0.81 ± 2.12	0.98 ± 2.34	0.78 ± 1.75	0.280^∧^

LOS: length of stay. SD: Standard Deviation. ^*∗*^Pearson Chi-Squared test. ^∧^One way ANOVA. Median LOS for all groups = 0.

**Table 5:** Types of complication/readmission reason.

	Normal weight	Overweight	Class I obesity	Class II obesity	Class III obesity	*Total*	*P* value
	BMI 18.5–24.9 (*n* = 280)	BMI 25.0–29.9 (*n* = 620)	BMI 30.0–34.9 (*n* = 438)	BMI 35.0–39.9 (*n* = 213)	BMI > 40.0 (*n* = 95)	All BMI (*n* = 1646)	
Abdominal collection	1	4	2	1	0	8	0.926
Bile leak requiring ERCP	0	1	1	0	0	2	0.880
Bile leak requiring hepaticojejunostomy	0	1	0	0	0	1	0.796
Constipation	0	2	0	0	0	2	0.502
Death	0	1	0	0	0	1	0.796
Post-op nausea	3	1	1	0	0	5	0.136
Missed pancreatic cancer	0	0	1	0	0	1	0.608
Nonspecific chest pain	0	3	1	0	1	5	0.568
Pain with no cause found	4	8	1	2	1	16	0.418
Port site bleeding	0	0	1	0	0	1	0.607
Retained stone	1	3	2	1	0	7	0.975
Small bowel obstruction	0	1	1	1	0	3	0.797
Urinary retention	0	2	1	0	1	3	0.666
Wound infection	0	4	3	2	0	9	0.514
Total	9	31	15	7	3	65	0.183
*Complication total/BMI category number*	3.21%	5.00%	3.42%	3.29%	3.16%	3.95%	

BMI: Body Mass Index, ERCP: Endoscopic Retrograde Cholangiopancreatography; *P* value: all statistical tests using Pearson Chi-Squared unless stated.
